# Exploring Sound Frequency Detection in the Golden Rabbitfish, *Siganus guttatus*: A Behavioral Study

**DOI:** 10.3390/ani14202967

**Published:** 2024-10-14

**Authors:** Shenwei Zhang, Xuguang Zhang, Xianming Tang, Shouyu Zhang

**Affiliations:** 1 Engineering Technology Research Center of Marine Ranching, College of Marine Ecology and Environment, Shanghai Ocean University, Shanghai 201306, China; 13515722852@163.com (S.Z.); syzhang@shou.edu.cn (S.Z.); 2 Hainan Provincial Key Laboratory of Tropical Maricultural Technology, Hainan Academy of Ocean and Fisheries Sciences, Haikou 571126, China

**Keywords:** *Siganus guttatus*, sound stimulation, fish behavior, acoustic deterrent, phonotaxis

## Abstract

**Simple Summary:**

The Golden Rabbitfish’s consumption of seaweed can seriously harm seaweed beds. To address the issue of their grazing on seaweed beds, sound can be used to deter them. The behavioral reaction of the Golden Rabbitfish to sine wave pulses was examined in this study. According to the findings, the fish responded to low-frequency sine pulses the most, exhibiting a clear escape response. These results allow for future attempts to employ sound to deter Golden Rabbitfish and present a theoretical strategy and data base.

**Abstract:**

This study investigates the auditory capabilities of Golden Rabbitfish (*Siganus guttatus*) and the potential efficacy of sound-based deterrent methods for behavior control. Behavioral experiments were conducted on Golden Rabbitfish to assess their responses to sound stimuli. Sinusoidal pulses in the range of 100~800 Hz, based on previous research on auditory evoked potentials (AEPs), were utilized. An analysis of behavioral trajectories, swimming speed, and acceleration changes revealed the fish’s reactions to varying frequency sound stimuli. The results indicate that Golden Rabbitfish exhibited increased swimming activity and speed when stimulated by sound and notably moved away from the source under prolonged exposure to low-frequency acoustic signals. Specifically, the fish displayed the most significant response to 200 Hz sinusoidal pulses with a response threshold of 113~126 dB. These findings suggest that Golden Rabbitfish are particularly sensitive to low-frequency noise, aligning with AEP study outcomes. This study concludes that employing sound stimuli to deter Golden Rabbitfish in practical settings holds promise for mitigating economic losses in seaweed farming due to Golden Rabbitfish grazing.

## 1. Introduction

The Golden Rabbitfish (*Siganus guttatus*) is a member of the genus *Siganus* within the family *Siganidae* of the order *Perciformes*. Widely distributed in the South China Sea and the East China Sea [1], this small coastal fish is highly sought after for consumption. The Golden Rabbitfish demonstrates a notable ability to thrive across a broad range of salinity levels and displays a strong affinity for various types of bait, making it a promising candidate for aquaculture endeavors [2,3]. Primarily herbivorous, it predominantly feeds on seaweed. In aquaculture practices, the Golden Rabbitfish is utilized to mitigate the need to manually clean nets by allowing it to consume algae adhering to net cages. Given its preference for consuming larger algae, such as *Sargassum*, the Golden Rabbitfish is frequently employed in co-cultivation to manage algae overgrowth and maintain water quality in aquaculture ponds [4,5,6]. In its natural marine habitat, the Golden Rabbitfish plays a crucial role as an herbivore in preserving the ecological balance of coral reef ecosystems [7]. Nonetheless, its grazing activities on seaweed beds can contribute to the decline in these habitats, resulting in diminished ecological functions and economic setbacks in seaweed aquaculture [8].

Sound plays a critical role in influencing marine animals [9]. Marine animals use sound to communicate within species as well as to understand and explore the ocean environment. Additionally, both biological and non-biological sounds prevalent in the aquatic environment significantly impact the survival and reproduction of fish [10,11]. The escalation of anthropogenic noise in aquatic ecosystems, driven by societal development and technological progress, is increasingly impacting the foraging and swimming behaviors of wild fish [12]. This noise can significantly alter the behavior and physiology of various fish species [13], leading to changes in their activities and responses due to sound variations. Consequently, leveraging fish behavioral responses to sound production offers a potential strategy for widespread fish deterrence [14]. Moreover, by considering fish phonotaxis, targeted sound stimuli can be utilized to manage Golden Rabbitfish, effectively deterring them from seaweed beds and reducing their algae grazing activities.

Currently, methods for determining auditory thresholds in fish mainly focus on behavioral and electrophysiological approaches. In the past, behavioral methods were primarily used to measure fish auditory capabilities, often involving electrical stimulation or food rewards. In contemporary research, electrophysiological techniques are predominantly utilized for assessing fish auditory thresholds [15,16]. Among these methods, auditory evoked potentials (AEPs) are widely acknowledged for their minimally invasive nature and efficiency in swiftly evaluating fish auditory sensitivity. While AEP measurements may indicate slightly higher auditory thresholds compared to behavioral assessments [14,16], discrepancies between thresholds obtained via AEPs and behavioral approaches within the same species have been noted [17,18,19]. Therefore, prior to implementing sound deterrent strategies for specific fish species, it is imperative to account for potential environmental influences. In a prior study, juvenile Golden Rabbitfish’s auditory sensitivity to acoustic frequencies spanning 100–800 Hz was assessed using AEPs [20]. Subsequently, based on the outcomes of the AEP investigation, acoustic frequencies within the 100–800 Hz range were selected to administer varying sound intensities to observe Golden Rabbitfish responses and any resultant behavioral alterations. By integrating the auditory thresholds of Golden Rabbitfish with the results of this study, valuable data supporting future sound deterrence initiatives are furnished.

## 2. Materials and Methods

### 2.1. Ethics Statement

Fish samples used for the purpose of this study were approved by the Animal Ethics Committee of Shanghai Ocean University. The experimental protocol for fish was performed in strict accordance with the requirements of Animal Ethics Procedures and Guidelines of the People’s Republic of China.

### 2.2. Study Species and Husbandry

A total of 150 Golden Rabbitfish with a total length in the range of 10.5~19.4 cm and an average weight in the range of 21.36~56.28 g were transported by air from Hainan to Shanghai. The experimental fish were temporarily housed in a circular recirculating water tank with a diameter of 120 cm. The water temperature was set at 25 °C and salinity controlled at 29~30‰ under normal natural light conditions. During the temporary housing phase, all the fish were fed artificial pellet feed every two days at random intervals. A week prior to the commencement of the experiment, individuals displaying robust vitality and lacking external injuries were specifically chosen and placed in a rectangular experimental tank (3.0 m × 1.0 m × 1.0 m with a water depth of 0.4 m) for acclimatization. The conditions maintained during this acclimatization period mirrored those of the circular tank, and the feeding site remained constant.

### 2.3. Acoustic Stimuli

The sound stimuli were generated using a waveform generator (Tektronix, AFG3021B, bandwidth 25 MHz, Tektronix China Ltd., Shanghai, China) and transmitted to underwater speakers (UW-30, Lubell Labs Inc., Columbus, OH, USA) via a power amplifier (D75A, Crown Audio, Elkhart, Indiana, UK). The underwater speakers were positioned at the right end of the rectangular experimental tank and separated from the experimental fish using a barrier net to prevent the fish from swimming out of the experimental area during the experiment. The applied sound stimuli were set as sine pulses with frequencies including 100, 200, 300, 400, 600, and 800 Hz. The sound stimuli were generated using a waveform generator, and the volume of the sound played by the underwater speaker was controlled by setting different output voltages on the waveform generator. The entire experiment used five different levels of sound stimuli. The output voltages of the waveform generator, from lowest to highest, were 100, 150, 200, 250, and 300 mVpp.

### 2.4. Sound Field Measurement

**Figure 1:**
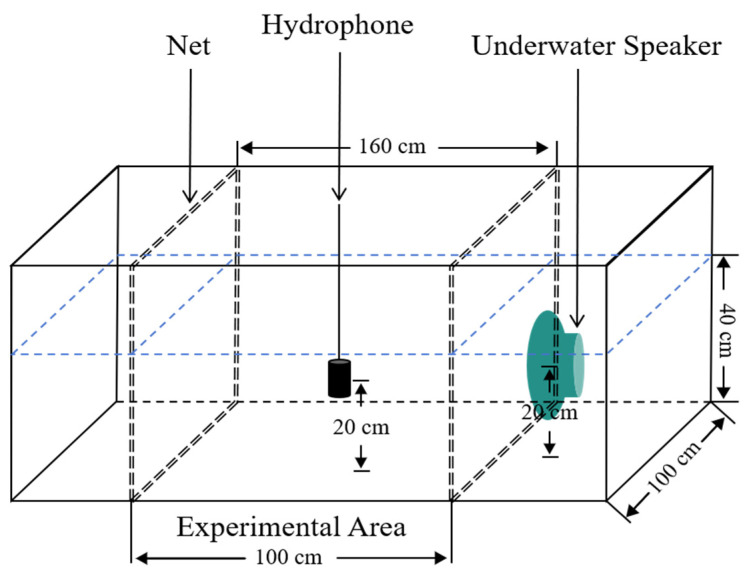
Sound field measurement diagram.

In this study, a section of a rectangular experimental water tank (1.6 m × 1.0 m × 1.0 m) was selected as the experimental area. The area was enclosed with nets at the front and back to prevent the experimental fish from swimming out during the experiment. It was evenly divided into 48 smaller sections in a planar layout for underwater sound field measurements. Before the measurements, the oxygenation and heating equipment were turned off to maintain silence and prevent interference from equipment noise. For each underwater sound field measurement, a hydrophone (Reson TC4032, Teledyne Marine, Slangerup, Denmark; sensitivity −170 dB re 1 V/μPa) was positioned at the center of each designated small section, with the measurement depth being approximately 20 cm above the bottom of the tank (the middle of the water column) (Figure 1). The hydrophone was connected to an analog-to-digital converter (Reson EC6073, Teledyne Marine, Slangerup, Denmark) and continuously powered by a battery. The signal from the converter was transmitted to a data acquisition system (3050-A-040, Brüel & Kjær, Skodsborgvej, Denmark) and then transferred to computer software for analysis. After the measurement started, the sound was played continuously until the hydrophone completely recorded the sound at each playback. The sound at each point was played for at least 1 min to ensure the hydrophone could continuously record underwater sounds for 20 s. Each sound level at all points was measured three times and averaged to eliminate interference. The data collected from all measurements were analyzed and processed using BK Connect software (Version 2019, Brüel & Kjær, Skodsborgvej, Denmark) to obtain the averaged underwater sound pressure level for each region, and an underwater sound field heatmap was generated.

### 2.5. Experimental Design: Sudden Sounds

**Figure 2:**
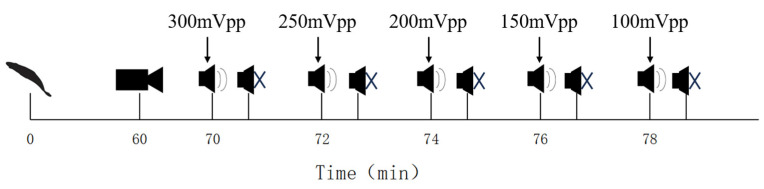
Single experiment procedure diagram.

Five uninjured Golden Rabbitfish were selected as experimental subjects and placed into the experimental area simultaneously. To minimize interference, the oxygen pump, heating rod, and other equipment in the experimental tank were disconnected an hour before the experiment. The lights remained on throughout to ensure consistent lighting conditions. An experimental camera (DS-2SC1Q120lY-TE, Hikvision, Hangzhou, Zhejiang, China) was positioned above the experimental area and secured with a bracket. Before the experiment began, the camera was adjusted to capture the entire experimental area. The experimental timeline is illustrated in Figure 2.

The camera was activated for recording after one hour of fish acclimation, followed by the application of acoustic stimulation ten minutes later. Each sound stimulus, lasting 10 s, was succeeded by a 1 min 50 s period of silence to allow the fish to acclimate. A single experimental process is defined as the playback of sound stimuli at the same frequency, with the order of sound stimulation decreasing in descending order: 300, 250, 200, 150, and 100 mVpp. After each set of frequency experiments, the oxygenation pump, heating rod, and other equipment were restarted to maintain the experimental fish in good condition for 1 h. Following this period, there was an additional 1 h quiet adaptation phase before starting the next set of frequency sound stimulus experiments. During the experiment, the initial positions of each experimental fish were recorded before each sound playback. These positions were recorded according to the 48 designated areas outlined in the underwater sound field measurements. Each set of experiments was repeated three times. The sequence of experiment repetitions began after completing all groups. The sequence of sudden sound stimulus frequencies applied throughout the experiment is 100, 200, 300, 400, and 600 Hz. After each experiment group, the experimental fish were allowed to acclimate in the temporary housing environment.

### 2.6. Experimental Design: Acoustic Stimulation at Different Frequencies

**Figure 3:**
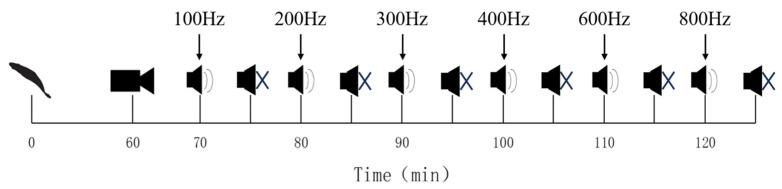
A diagram of the experimental procedure for continuous noise at different frequencies.

Five uninjured Golden Rabbitfish were selected as experimental subjects and placed into the experimental area simultaneously. The experimental methods were similar to those used in the previous experiment. Prior to the experiment, the fish were acclimated in a silent environment for 1 h by deactivating equipment such as the oxygen pump and heating rod. The experiment was designed to sequentially play continuous sinusoidal pulses at frequencies of 100, 200, 300, 400, 600, and 800 Hz. Prior to each new frequency’s sound stimulus, there was a continuous 5 min silent buffering period. The sound levels chosen for the experiment were consistently set at the maximum voltage output of the waveform generator (300 mVpp). Each sound stimulus lasted for 5 min, followed by a 5 min silent adaptation period during which the oxygenation pump and other equipment remained off to eliminate human interference. After the silent period, the next frequency’s sound stimulus was played. This process continued until the 800 Hz sound stimulus was completed (Figure 3). Throughout the experiment, the lights were kept on to ensure consistent lighting conditions and to eliminate any potential interference caused by variations in illumination.

### 2.7. Behavioral Data Processing

The experiment was recorded on video, which was subsequently edited and standardized for frame rate. Fish behavior was analyzed using the behavior analysis software Lolitrack v5. This analysis enabled the quantification of various behaviors exhibited by the Golden Rabbitfish, including swimming speed, acceleration, and positions at each frame. For the sudden sound stimulus experiments, approximately 70 s of video footage before and after the stimulus was chosen for further analysis. The swimming speed data for each fish at each frame were integrated and averaged using Python (version 3.12.3, 64-bit) for subsequent analysis of results. The control group was selected from a random period of silence before the sound stimulus started. The duration of the control group video was similar to the analysis period of the sound stimulus, approximately 70 s. For the continuous sound stimulus experiment, the control group was selected similarly to the previous experiment. A random period during the silent phase was chosen as the control, lasting 5 min, which is the same as the duration of the continuous sound stimulus. Trajectories and swimming speeds were analyzed from the video. Additionally, the behavioral trajectories of the Golden Rabbitfish were plotted using Origin (2019b, 64-bit).

### 2.8. Statistical Analysis

In this study, we investigated the behavioral responses of fish to various sound treatments, assessing their reactions before, during, and after exposure. The sound treatment was treated as a fixed factor in our analysis. We conducted a one-way repeated measures analysis of variance (one-way RM ANOVA) to identify any significant differences. If variances were detected, we carried out post hoc tests (Holm–Sidak test) to examine specific intergroup variations with a significance level set at 0.05. All results are presented as mean ± standard error (x ± SE). Statistical analysis was performed using SPSS software 26.0 (IBM Corp., Armonk, NY, USA).

## 3. Results

### 3.1. Acoustic Distribution

**Figure 4:**
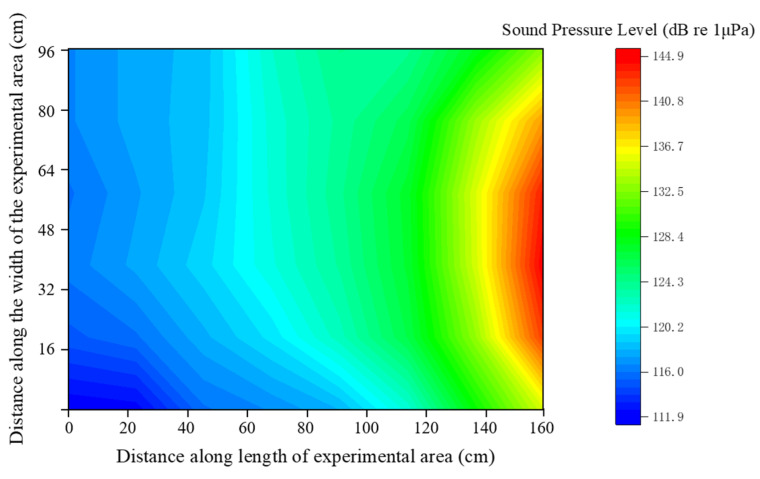
The underwater sound field when the waveform generator outputs a voltage of 300 mVpp.

The underwater sound intensity exhibits an overall gradient change. As the distance from the loudspeaker increases, the sound pressure level in the underwater sound field diminishes. When the waveform generator outputs voltages of 100, 150, 200, 250, and 300 mVpp, the maximum sound pressures produced by the underwater speaker are 136.5, 141.3, 143.2, 144.3, and 144.9 dB, respectively. Across the entire experimental area, the minimum underwater sound pressures are 100.8, 106.1, 108.7, 111.3, and 111.9 dB, respectively. However, due to the reflection of sound by the tank walls, the underwater sound pressure is slightly higher in some regions on the far left of the experimental area compared to the inner side (Figure 4). The overall underwater sound field in the experimental area generally shows a gradient increase from left to right.

### 3.2. The Response of S. guttatus to Sudden Sounds

**Figure 5:**
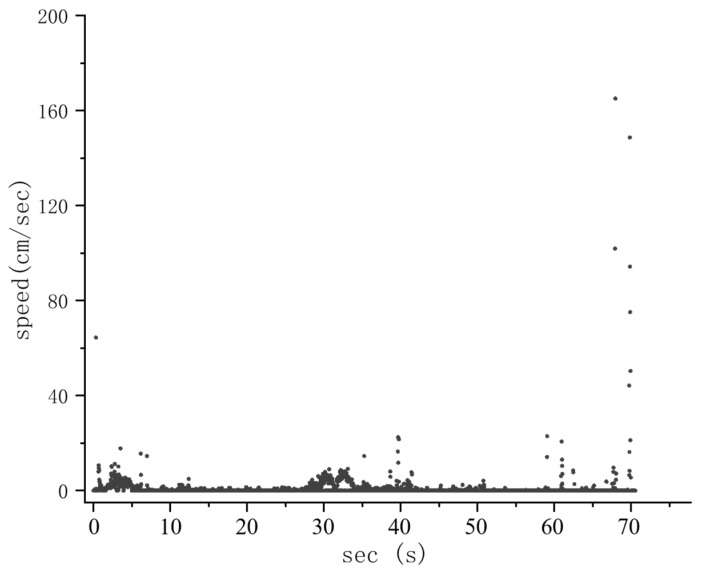
Distribution of swimming speeds of five *S. guttatus* during control group experiment.

**Figure 6:**
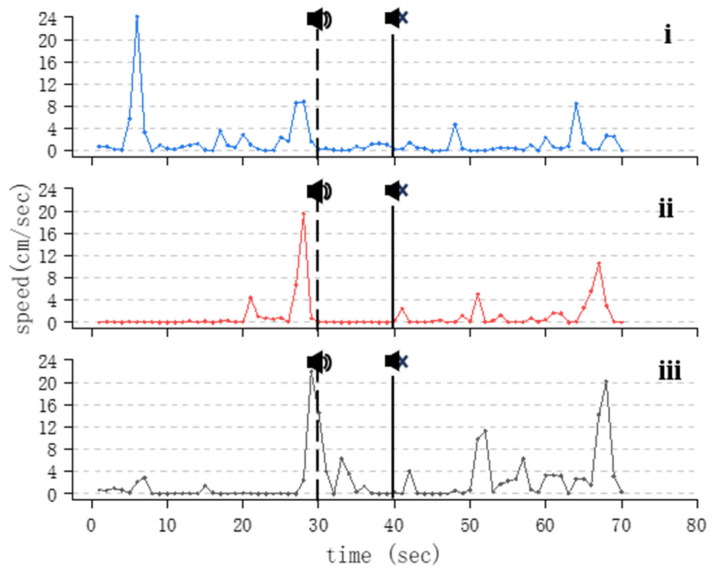
Changes in swimming speed of *S. guttatus* under 200 Hz acoustic stimulation. Subfigures (**i**–**iii**) represent maximum sound stimuli applied at 144.9 dB, 143.2 dB, and 136.5 dB.

In the control group where no sound stimulus was present, the fish predominantly displayed slow swimming or stationary behavior. Large-scale random swimming events were infrequent, with only 2–3 occurrences being observed throughout the observation period, and the overall swimming speed exhibited a random distribution (Figure 5). During the entire experiment, in the experimental groups exposed to 100 Hz and 200 Hz sounds on treated fish, it exhibited a notably lower overall swimming speed during the sound playback phase (30~40 s) in comparison to the speed when no sound stimulus was played. Following the sound playback, the average swimming speed of the five treated fish was significantly lower than that before the sound stimulus was applied. However, after the sound stimulus ended, the fish showed more swimming behavior compared to before, showing noticeable increases in the frequency and amplitude of speed changes. These responses were particularly prominent in response to the 200 Hz group (Figure 6).

**Figure 7:**
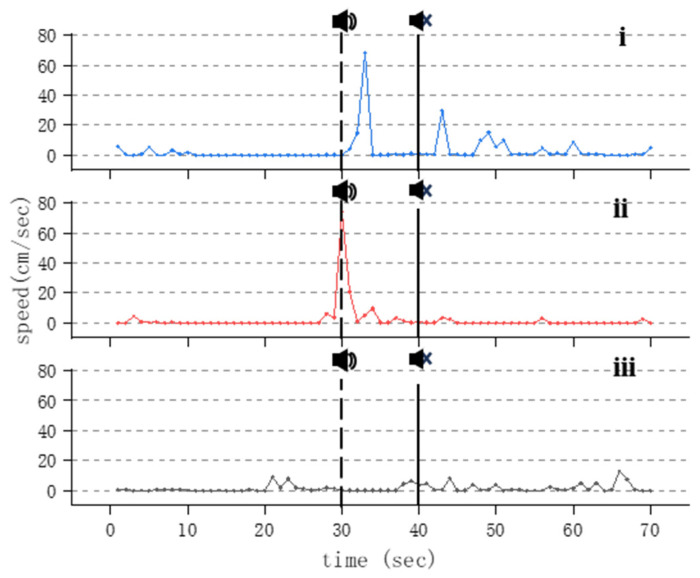
Changes in swimming speed of *S. guttatus* under 400 Hz acoustic stimulation. Subfigures (**i**–**iii**) represent maximum sound stimuli applied at 143.2 dB, 141.3 dB, and 136.5 dB.

As the frequency of the applied sound increases, when the acoustic stimulation frequency reaches 300~400 Hz, the magnitude of swimming behavior of Golden Rabbitfish under the influence of sound increases. That is, after acoustic stimulation, the maximum swimming speed of the experimental fish exceeds 500 pixels/s. Additionally, after acoustic stimulation, the swimming speed of the treated fish increases significantly and exhibits noticeable fluctuations compared to before the acoustic stimulation. This phenomenon persists when the acoustic stimulation frequency is at 300 Hz until the lowest acoustic stimulation intensity is applied. When the frequency reaches 400 Hz, this phenomenon continues until the maximum applied sound reaches 141.3 dB (the output voltage of the waveform generator is 150 mVpp) (Figure 7).

**Figure 8:**
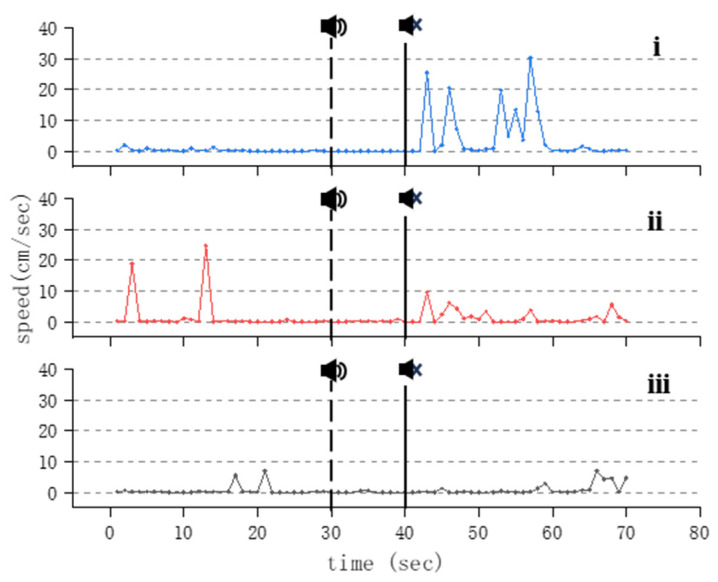
Changes in swimming speed of *S. guttatus* under 600 Hz acoustic stimulation. Subfigures (**i**–**iii**) represent maximum sound stimuli applied at 144.9 dB, 144.3 dB, and 143.2 dB.

When exposed to a stimulating sound frequency in the range of 600~800 Hz, the swimming activity of the fish under investigation diminishes, with the swimming speed predominantly being at a standstill during the sound exposure period. However, following the cessation of stimulation, notably in the groups subjected to sound stimuli of a higher intensity, there is a significant surge in swimming speed among the treated fish, accompanied by a marked increase in the frequency of swimming behaviors (Figure 8). Notably, throughout the duration of the experiment, the behavioral response of the treated fish is evident in groups exposed to sound pressure levels of 144.9 dB and 144.3 dB (corresponding to output voltages of 300 mVpp and 250 mVpp, respectively) when the acoustic stimulation frequency is set at 600 Hz. Conversely, at an acoustic stimulation frequency of 800 Hz, this behavioral response is solely observed in the group exposed to a maximum sound pressure of 144.9 dB.

This study examines the behavioral responses of Golden Rabbitfish to acoustic stimulation across a range of frequencies. The fish in the experimental group exhibited varying sensitivities to different sound frequencies. At 100 Hz, responses were observed only to a maximum sound pressure of 143.2 dB (generated by an output voltage of 200 mVpp). The most pronounced responses were recorded at 200 Hz and 300 Hz, with fish reacting to the lowest sound intensity. At 400 Hz, a sound pressure of 136.5 dB or higher (generated by an output voltage of 100 mVpp) elicited responses. The fish also responded to 600 Hz with a maximum sound pressure of 144.3 dB (generated by an output voltage of 250 mVpp). However, at 800 Hz, responses were only observed for the maximum acoustic stimulation level.

**Figure 9:**
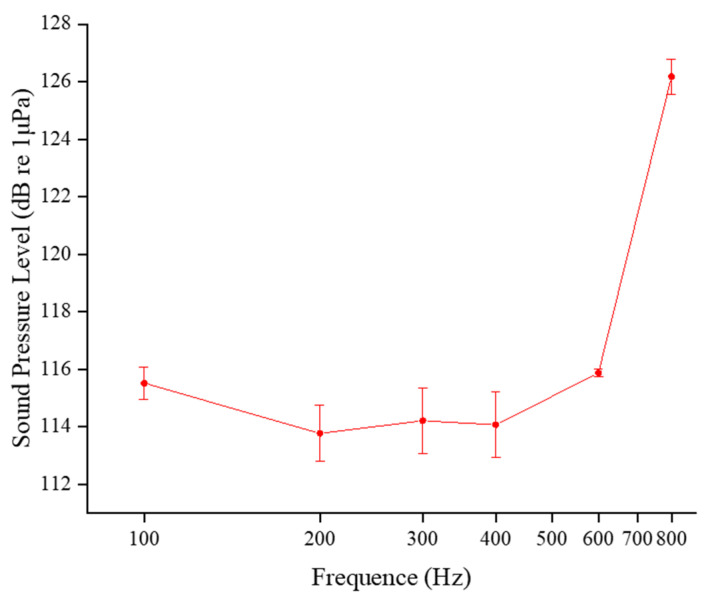
The response of *S. guttatus* to acoustic stimulation at different frequencies.

By correlating sound field heat maps with experimental videos and analyzing sound levels at the locations of the treated fish during acoustic stimulation, response intensity maps were generated for various acoustic stimuli at different frequencies (Figure 9). The response pattern of Golden Rabbitfish exhibits a V-shaped curve: at lower frequencies, the fish respond to smaller sound stimuli, with a minimum threshold of 113.78 ± 0.98 dB at 200 Hz. The species displays heightened sensitivity to acoustic stimulation within the frequency range of 200~400 Hz, with an average response sound level ranging from 113 to 115 dB. In response to high-frequency acoustic stimuli, the sound level reaction of Golden Rabbitfish peaks at nearly 116 dB at 600 Hz and 126.18 ± 0.61 dB at 800 Hz.

### 3.3. The Response of S. guttatus under Continuous Acoustic Stimulation at Different Frequencies

**Figure 10:**
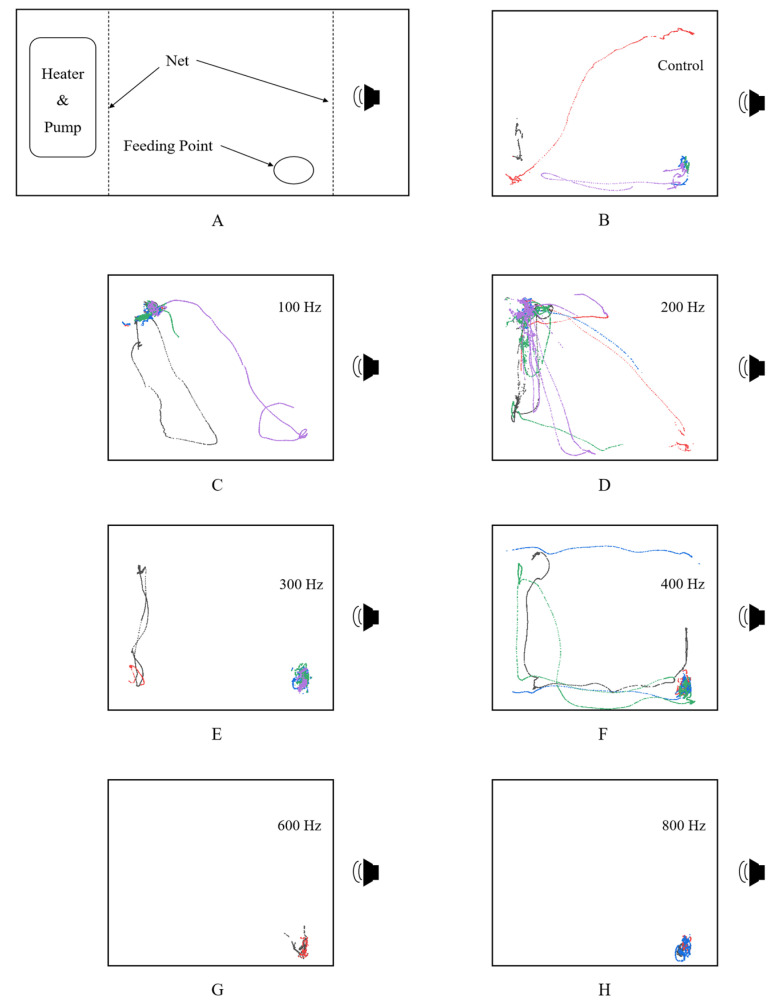
The behavior trajectory of *S. guttatus* under continuous sound stimulation. ((**A**): Diagram of experimental area; (**B**): no acoustic stimulation applied; (**C**–**H**): acoustic stimulation applied at frequencies of 100, 200, 300, 400, 600, and 800 Hz, respectively).

When exposed to sounds of the same magnitude, the behavior of the treated fish varies in degree depending on the frequency of the sound, mainly manifested as a tendency to move away from the speaker at lower acoustic frequencies (Figure 10).

When no sound is played, the swimming activity of the fish is irregular, and they tend to randomly swim throughout the entire experimental area, but they often stay in the bottom right corner of the experimental area (Figure 10B), which may be due to the proximity of the feeding point in that location. When exposed to the maximum sound level of 144.9 dB (the output voltage of the waveform generator is 300 mVpp), the fish show a noticeable tendency to move away from the source of sound at frequencies of 100 Hz and 200 Hz, with the effect being more pronounced at 200 Hz, where all of the fish swim to the far-left end of the experimental area. At ambient sound frequencies of 300 Hz and 400 Hz, the “escape” response is not prominent, with only certain treated fish exhibiting this behavior, while others tend to stay close to the feeding area. Conversely, at 600 Hz and 800 Hz, no distinct escape behavior is noted, and there is a lack of significant swimming activity among the fish.

**Figure 11:**
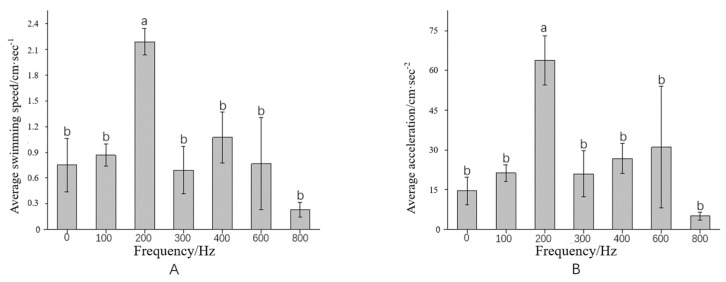
The behavior response changes in *S. guttatus* under continuous sound stimulation. (**A**) shows the average swimming speed; (**B**) shows the average acceleration. The letter (a) denotes a significant difference (*p* < 0.05), a letter (b) denotes a non-significant trend (0.05 < *p*).

The speed and acceleration data of fish exposed to five minutes of continuous sound were analyzed. Golden Rabbitfish exhibited the most significant response to a 200 Hz sound frequency (*p* < 0.05) under the same acoustic intensity (Figure 11). A correlation was observed between the average swimming speed of the treated fish and the sound frequency during continuous acoustic exposure. Apart from the 200 Hz group, the swimming speeds of experimental groups were similar to that of the control group. The highest average swimming speed was recorded in the 200 Hz group, showing a significant difference compared to the other groups (P_speed_ = 0.01; F = 6.054). In contrast, the experimental group exposed to 800 Hz had a significantly lower average swimming speed than the control group, but the difference was not significant (*p* > 0.05). A similar pattern was observed for acceleration: the 200 Hz group had the highest average acceleration with a significant difference (P_acceleration_ = 0.01; F = 6.326), while the other groups had higher accelerations than the control group but without significant differences (*p* > 0.05).

## 4. Discussion

Various fish species demonstrate diverse responses to noise exposure. Zebrafish (*Danio rerio*), for example, typically display startled reactions and an initial increase in swimming speed when exposed to noise, followed by a notable decrease in speed with prolonged exposure. In contrast, European minnows (*Phoxinus phoxinus*) initially exhibit a decrease in swimming speed in response to noise, which then rapidly increases over time [21,22]. The behavioral changes in Golden Rabbitfish are akin to those of zebrafish and European minnows. Following sudden sound stimuli, Golden Rabbitfish generally experience a reduction in swimming speed, particularly pronounced under low-frequency sound influences. We speculate that this decrease in speed may facilitate information transmission among Golden Rabbitfish, allowing for a more accurate assessment of environmental threats similar to that of other fish [22,23].

**Figure 12:**
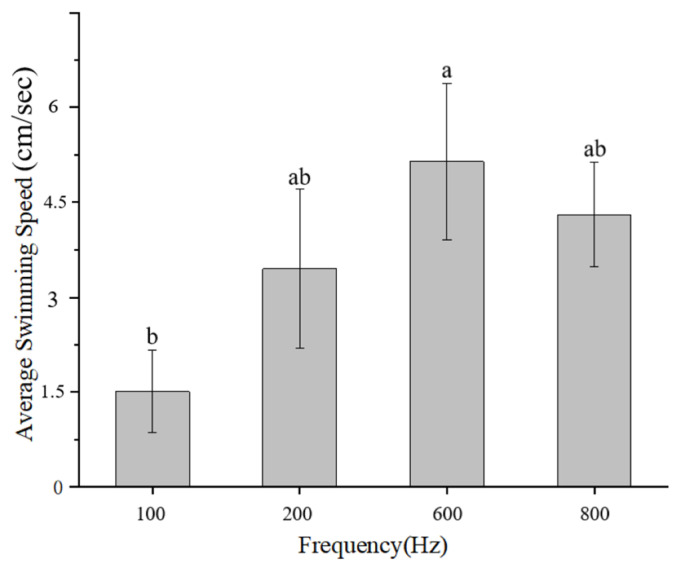
The average swimming speed of *S. guttatus* after sudden sounds at the same level. The letter (a) denotes a significant difference (*p* < 0.05), a letter (b) denotes a non-significant trend (0.05 < *p*).

As the acoustic frequency increases, Golden Rabbitfish demonstrate a gradual escalation in swimming activity, particularly evident in the acceleration in swimming speed (Figure 7). This response is likely attributed to a startle reaction induced by the intensity of sound within the specific frequency range. Following the abrupt cessation of acoustic stimuli, the fish display a notable increase in swimming speed (Figure 6, Figure 7i and Figure 8i,ii). This heightened behavior is most prominent after exposure to high-frequency sounds, with significant variations being observed in the speed of Golden Rabbitfish post-sound cessation at 600 Hz (*p* = 0.024; F = 2.292), which are generally higher than the average swimming speed of fish treated under normal conditions (Figure 11B and Figure 12). Throughout the sudden sound experiment, the swimming speed of the treated fish tended to exhibit a V-shaped pattern, with increased activity before and after sound application and decreased activity during acoustic application. This pattern mirrors the behavioral alterations seen in Atlantic cod (*Gadus morhua*) when subjected to continuous sound, characterized by decreased activity levels and an increased swimming depth during noise exposure, although depth changes were not quantifiable in this study.

After analyzing and comparing the behavioral responses of the treated fish during the sudden sound experiment, it was found that there is a minimum sound threshold that elicits a response from the fish at different frequencies. This means that Golden Rabbitfish will only respond to sounds above a certain level; a lower sound level indicates that the fish are more sensitive to that frequency. The results show that Golden Rabbitfish are the most sensitive to lower-frequency sounds, with their maximum sensitivity occurring at 200 Hz. This aligns with previous studies that used electrophysiological methods to study the auditory sensitivity of juvenile Golden Rabbitfish [20]. Additionally, the response curve of the experiment’s results is similar to the auditory threshold curve seen in previous experiments. However, there are some differences when comparing the auditory thresholds measured by electrophysiological methods. The results of this experiment suggest that Golden Rabbitfish respond to lower frequencies at slightly higher sound levels than the auditory threshold levels measured by electrophysiological methods; on the other hand, at frequencies of 600 Hz and above, they respond at much lower sound levels compared to the electrophysiological measurements. These discrepancies may be due to changes in the auditory abilities of adult Golden Rabbitfish as they grow and mature. It is known that individual variations can affect the auditory capabilities of fish, with age and size playing roles in the sensitivity to sound [24]. Similar to zebrafish, which experience changes in auditory thresholds as they mature and age, Golden Rabbitfish may also exhibit variations in their auditory responses [25]. Simultaneously, the auditory thresholds measured by electrophysiological methods are precise physiological data, focusing on a subset of the many functional factors that determine behavior (sensation and nerves), and the results measured by behavioral methods are closer to the natural environment, reflecting the fish’s responses to sounds in their actual surroundings [25]. The sound levels in the experimental environment can also impact the auditory perceptions of the fish, potentially leading to deviations in the experimental results obtained through behavioral methods.

Golden Rabbitfish demonstrate negative phonotaxis, displaying heightened sensitivity to frequencies ranging from 100 to 400 Hz as indicated by two auditory threshold measurement experiments. When exposed to low-frequency sounds (100–200 Hz) over an extended period, the fish consistently moved away from the sound source, as evidenced by the notable behavioral changes observed (Figure 10C,D). Significant differences in the average swimming speed and acceleration of Golden Rabbitfish were particularly notable during exposure to 200 Hz of sound compared to other frequencies (Figure 11), emphasizing their strong aversion to low-frequency sounds. In contrast, behavioral responses to frequencies in the range of 300–400 Hz varied among the experimental fish, with only some displaying a tendency to move away from the sound source (Figure 10E,F), while the others remained in close proximity. This suggests that not all fish exhibited escape reactions to sounds in this frequency range under the experimental conditions, possibly due to differences in perception or individual variability. However, not all fish exposed to prolonged sound stimuli exhibit behavioral changes such as distancing themselves from the sound source [26]. Studies have shown that zebrafish and Lake Victoria cichlids (*Haplochromis piceatus*) do not display a clear tendency to move away from sustained white noise or irregular intermittent pulses [21]. In contrast, the Convict cichlid (*Amatitlania nigrofasciata*) shows avoidance behavior in reaction to boat noise [27], while the Round goby (*Neogobius melanostomus*) tends to approach rumbling sounds [28]. These varied responses are attributed to the distinct phonotactic behaviors observed among different fish species. Fish demonstrate diverse phonotactic responses to sounds of the same frequency and differing sound pressures, with individual species also showing varying reactions to different sound types [29]. Most fish species exhibit negative phonotaxis, i.e., they tend to move away from sound sources in response to predator feeding sounds or anthropogenic noises such as ship noises [30]; despite this, they show positive phonotaxis whereby they are inclined to approach sounds produced by individuals of the same species, such as swimming noises or mating calls from the opposite sex [31]. Simultaneously, fish respond to various deterrent sounds by exhibiting variable degrees of avoidance at the same time. Li compared the avoidance responses of juvenile silver carp (*Hypophthalmichthys molitrix*) to four different sounds by playing the roar of an alligator, artificial sweep frequency, a piling noise, and a ship horn noise to analyze their escape and avoidance behaviors. The results show that juvenile silver carp exhibited a more significant avoidance response to the predator sound (alligator roar) and artificial sound (0–2000 Hz sweep-up frequency) than to industrial noise (piling sound) and ship noise (white noise); thus, the fish were more deterred by predator sounds than by artificial sounds [32].

The phonotactic behavior of fish can be harnessed to control their movements [18,33]. The outcomes of this study align with the anticipated efficacy of sound as a deterrent for Golden Rabbitfish. A crucial requirement for using sound to deter Golden Rabbitfish is that the species can perceive the sound frequency and respond behaviorally to it [14,34]. Adam et al. effectively deterred European eels (*Anguilla anguilla*) near a hydroelectric power station intake using low-frequency sound at 12 Hz [35]. In a fishing context, Turkey successfully deterred small cetaceans while targeting large turbot using an innovative acoustic trawl [36]. In addition, acoustic deterrence is also influenced by the sound pressure level. Fish respond to sound more behaviorally as the sound pressure level rises. For example, silver carp show 11 noticeable reactions to a 145 dB sound within 5 min, while in the same amount of time, they exhibit 20 discernible behavioral responses to a 155 dB sound [37]. The findings of this study reveal that Golden Rabbitfish display a distinct avoidance reaction when exposed to prolonged sinusoidal pulses at 100 and 200 Hz, approaching only after the sound ceases. This underscores the potential of using 100 and 200 Hz sinusoidal pulses for acoustically deterring Golden Rabbitfish. However, practical applications must account for the compounded effects of ambient noise in the natural setting. Additionally, fish may demonstrate adaptive responses to prolonged noise exposure, potentially showing decreased reactions over time, indicating a level of habituation. Some fish may gradually acclimate to sound stimuli, which is linked to their tolerance levels [33,38]. Given the significant variability in response levels and behavioral changes across fish species, when implementing acoustic deterrence in natural aquatic settings, the potential impact on other species should be considered, including their physiological stress responses [39,40]. Furthermore, investigating the auditory thresholds of other fish species within the same marine environment is essential for a thorough selection of appropriate sound stimuli.

## 5. Conclusions

Sound significantly influences fish behavior, and it is quite feasible to control or influence their actions through various auditory stimuli. Fish exhibit different tendencies to be attracted to sounds, with their responses differing based on sound frequency and intensity. Golden Rabbitfish are highly sensitive to low-frequency sine pulses, with a lower auditory threshold at these frequencies. Additionally, prolonged exposure to sine pulses can lead to avoidance and escape behaviors, indicating a clear negative sound attraction and a negative response to certain sounds. By leveraging this characteristic of Golden Rabbitfish, it becomes more feasible to use sound as a means to drive them away over large areas.

## Data Availability

Data are contained within the article.
